# Supplementary material for: Mechanism of phosphate sensing and signaling revealed by rice SPX1-PHR2 complex structure
Source: Nat Commun. 2021 Dec 2;12:7040. doi: 10.1038/s41467-021-27391-5 (PMC8639918; doi:10.1038/s41467-021-27391-5)
Supplement: Supplementary file 1 — Supplementary Information [file 41467_2021_27391_MOESM1_ESM.pdf]

Supplementary Information for "Mechanism of Phosphate Sensing and Signaling Revealed by Rice SPX1-PHR2 Complex Structure"

Jia Zhou[1,2,7], Qinli Hu[1,2,7], Xinlong Xiao[1], Deqiang Yao[3], Shenghong Ge[1,2], Jin Ye[4], Haojie Li[1], Rujie Cai[5], Renyang Liu[1], Fangang Meng[6], Chao Wang[4], Jian-Kang Zhu[1], Mingguang Lei[1*], Weiman Xing[5*]

[1]Shanghai Center for Plant Stress Biology and Center of Excellence in Molecular Plant Sciences, Chinese Academy of Sciences; Shanghai 200032, China.

[2]University of Chinese Academy of Sciences, Beijing 100049, China.

[3] State Key Laboratory of Oncogenes and Related Genes, Ren Ji Hospital, Shanghai Jiao Tong University School of Medicine, Shanghai 200127, China.

[4]MOE Key Laboratory for Membrane-less Organelles & Cellular Dynamics, Hefei National Laboratory for Physical Sciences at the Microscale, School of Life Sciences, Division of Life Sciences and Medicine, University of Science and Technology of China; Hefei 230027, China.

[5]Shanghai Key Laboratory of Plant Molecular Sciences, College of Life Sciences, Shanghai Normal University; Shanghai 200234, China.

[6]Beijing Neurosurgical Institute, Beijing Tiantan Hospital, Capital Medical University; Beijing 100070, China.

[7]These authors contributed equally: Jia Zhou and Qinli Hu

[*]Corresponding author. Email: Weiman Xing (weimanxing@shnu.edu.cn) and Mingguang Lei (mglei@cemps.ac.cn)

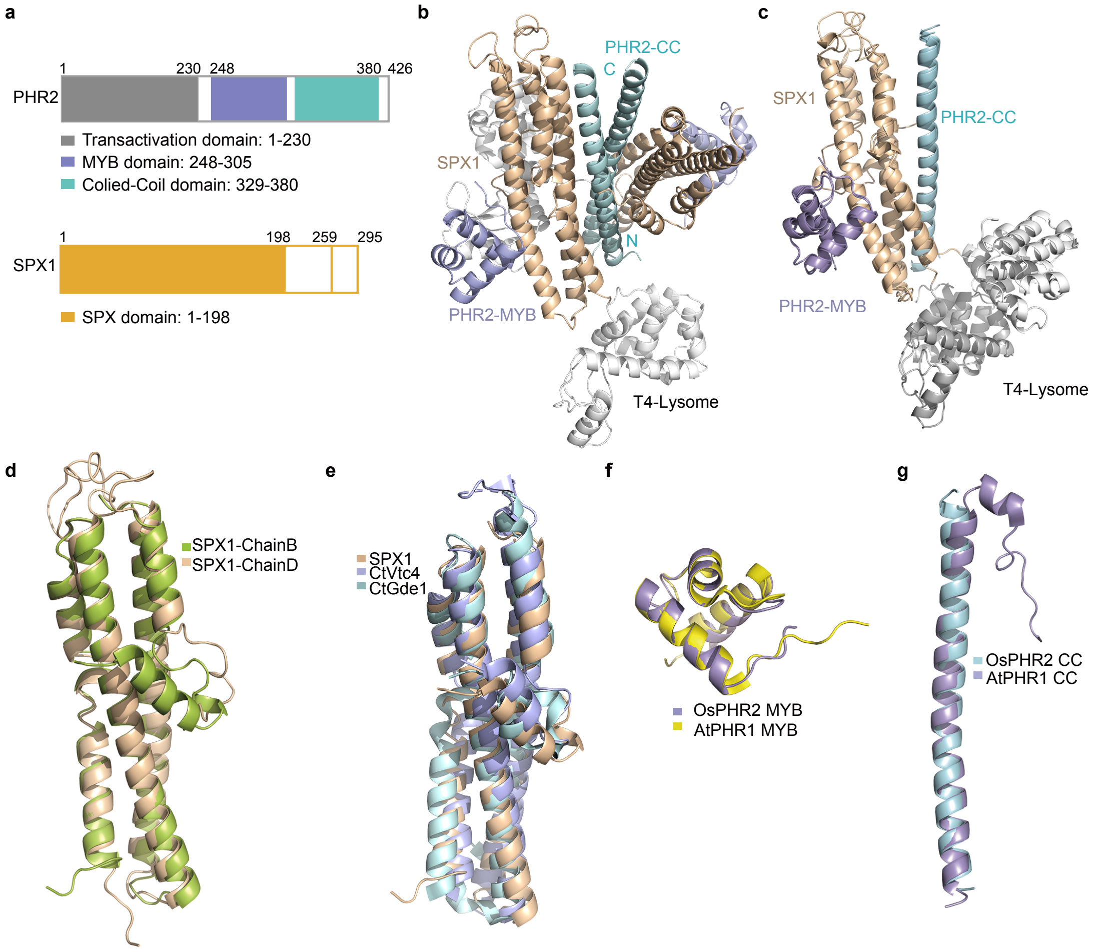

Supplementary Fig. 1 Both SPX1 and PHR2 structures are conserved.

a Domain architecture of SPX1 and PHR2 proteins. **b** Two molecules of T4L-tagged SPX1-PHR2 complex in the asymmetric unit were shown in cartoon representation. **c** Structural superimposition of two T4L-tagged SPX1-PHR2 complex molecules in the asymmetric unit. **d** Structural superimposition of two SPX1 molecules in the asymmetric unit. **e** Structural superimposition of SPX1 with other SPX-domain containing proteins, including CtGde1(PDB: 5IJJ) and CtVtc4(PDB: 5IJP). **f** Structural superimposition of rice PHR2 MYB (OsPHR2) and Arabidopsis MYB (AtPHR1, PDB: 6J4K). **g** Structural superimposition of rice PHR2 CC and Arabidopsis CC (AtPHR1, PDB: 6TO5).

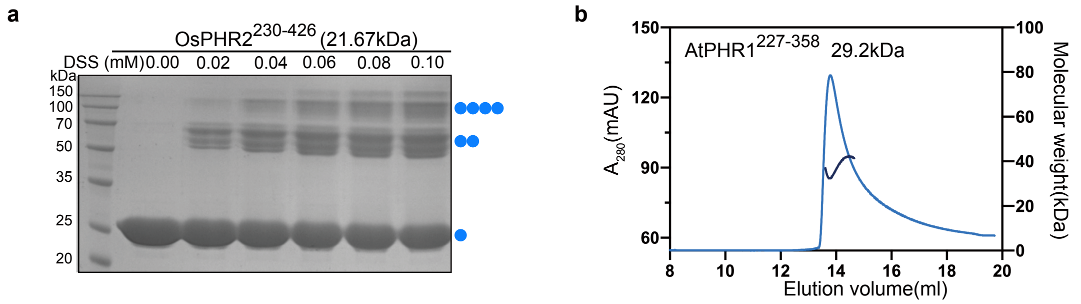

Supplementary Fig. 2 PHR2 primarily exists as a dimer in solution.

a Crosslinking of OsPHR2$^{230\text{-}426}$ with increasing concentrations of DSS (disuccinimidyl suberate). The oligomeric state of samples was analyzed by SDS-PAGE electrophoresis. The monomer, dimer and tetramer form of OsPHR2$^{230\text{-}426}$ were labeled by blue dots respectively. Experiments were independently repeated three times with similar results. Uncropped gel images are available as source data. **b** AtPHR1$^{227\text{-}358}$ is a dimer in solution based on the SEC-MALS analysis.

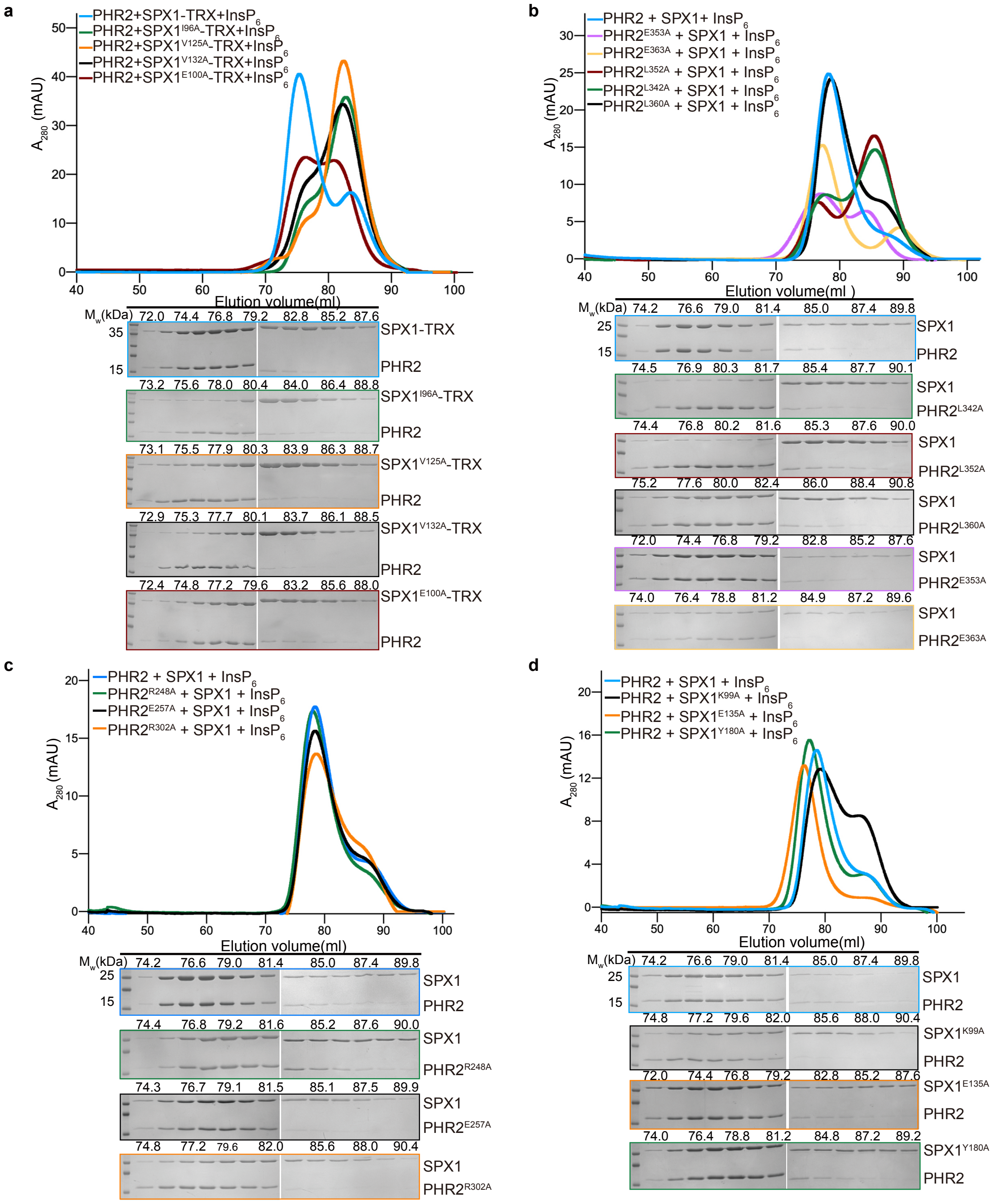

Supplementary Fig. 3 The effect of SPX1 and PHR2 mutations on the SPX1-PHR2 complex formation.

a, d Analysis of interactions between SPX1 mutations and wild type PHR2. $SPX1^{I96A}$, $SPX1^{V125A}$, $SPX1^{V132A}$, $SPX1^{K99A}$ and $SPX1^{E100A}$ disrupted the SPX1-PHR2 complex formation. $SPX1^{E135A}$ and $SPX1^{Y180A}$ had little effect on the SPX1-PHR2 complex formation. $SPX1^{I96A}$, $SPX1^{V125A}$, $SPX1^{V132A}$ and $SPX1^{E100A}$ were in the TRX-fused forms due to low expression level. **b, c** Analysis of interactions between PHR2 mutations and wild type SPX1. $PHR2^{R302A}$, $PHR2^{L352A}$, $PHR2^{L342A}$ disrupted the SPX1-PHR2 complex formation. $PHR2^{R248A}$, $PHR2^{R257A}$, $PHR2^{E353A}$, $PHR2^{E363A}$, and

PHR2^{L360A} had little effect on the SPX1-PHR2 complex formation. (Upper) Gel filtration profiles were color coded. (Lower) Coomassie-blue stained SDS-PAGE gels of peak fractions. The SEC assays were performed with HiLoad 16/600 Superdex 200 pg column. Experiments were independently repeated three times with similar results. Uncropped gel images are available as source data.

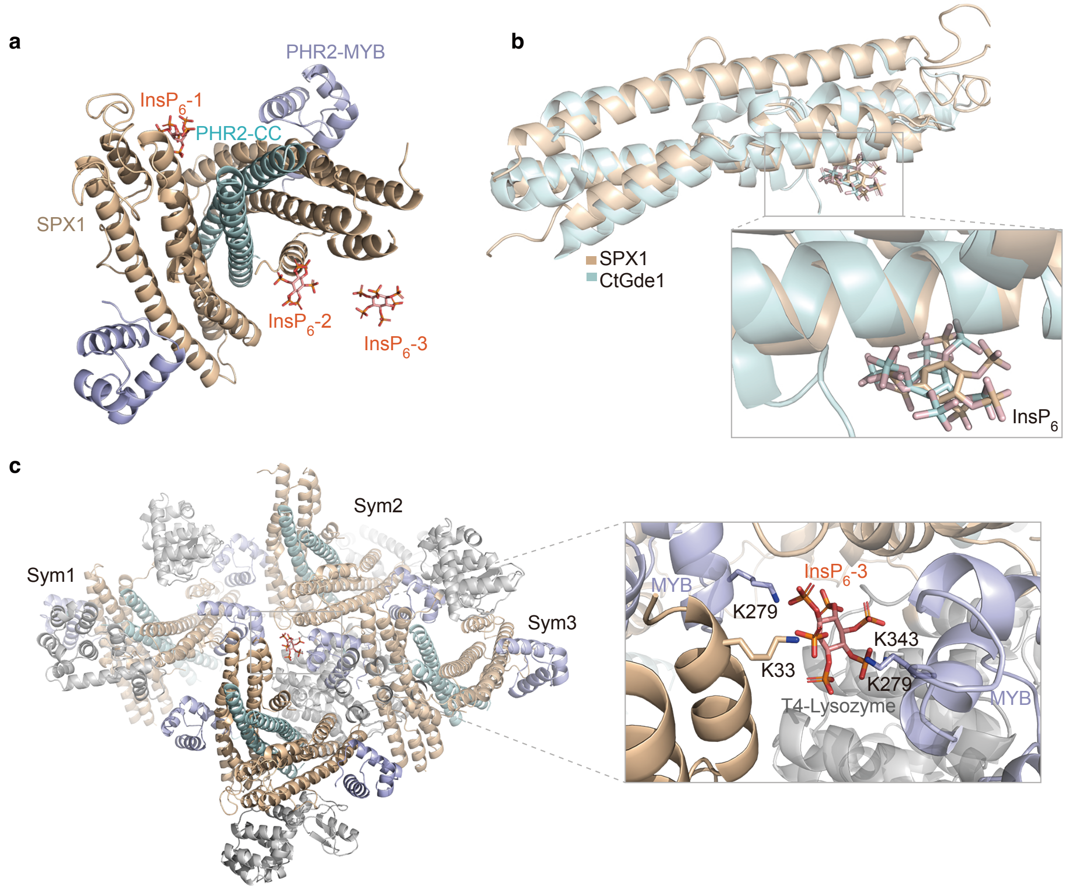

Supplementary Fig.4 InsP$_6$-binding mode is conserved in SPX domains.

a Two SPX1-PHR2 complex molecules in the asymmetric unit bind three InsP$_6$s. MYB and CC domains of PHR2 were colored in light blue and pale cyan respectively. SPX1 was colored in wheat. InsP$_6$s were shown in sticks and colored in red. **b** SPX domain-containing proteins share similar InsP$_6$ binding mode. Superimposition of SPX1 and CtGde1 (PDB: 5IJJ). Cartoon representations of SPX1 and CtGde1 were colored in wheat and pale cyan respectively. **c** The third InsP$_6$ molecule participated in crystal packing. The InsP$_6$ molecule interacted with SPX1 only through K33, but mainly interacted with two K279 residues of PHR2 MYB and K343 of T4L in sym1 and sym3 (sym: symmetry).

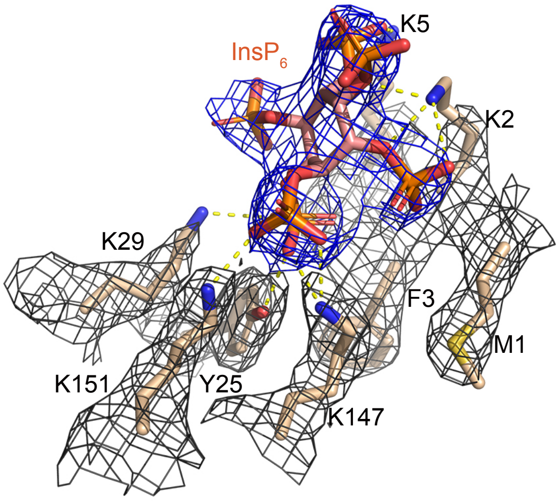

Supplementary Fig. 5 $InsP_6$ binding residues in SPX1 and $InsP_6$.
Close view of a stimulated annealing omit map (contoured at 1.1σ) around the $InsP_6$ in the SPX1. The $InsP_6$ is omitted to calculate the map.

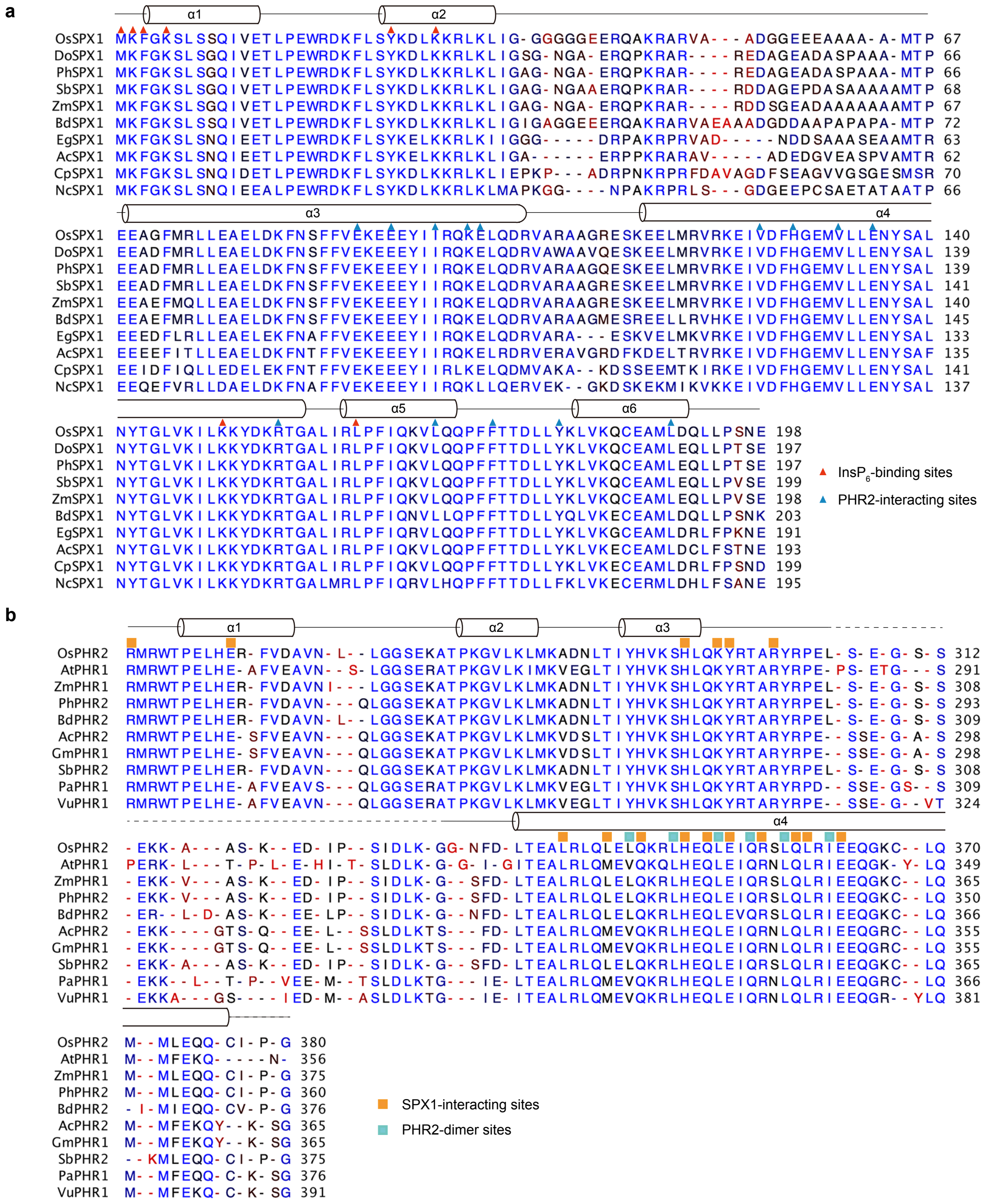

Supplementary Fig. 6 Sequence alignment of SPX domain-containing proteins and PHR proteins.

a Protein sequences of SPX domain-containing proteins from several species were aligned. The secondary structure elements of SPX domain were shown in the top. Highly conserved residues were colored in slate blue. The critical residues involved in InsPs binding and PHR2 interacting were displayed with red and blue trigons respectively. Species are: *Oryza sativa* (Os), *Arabidopsis thaliana* (At), *Dichanthelium oligosanthes* (Do), *Panicum hallii* (Ph), *Sorghum bicolor* (Sb), *Zea mays* (Zm), *Brachypodium distachyon* (Bd), *Elaeis guineensis* (Eg), *Ananas comosus* (Ac), *Carica papaya* (Cp). **b** Protein sequences of PHR from several species were aligned. The secondary structure elements of MYB and CC domains were shown in the top. Highly

conserved residues were colored in slate blue. The critical residues involved in SPX1 interacting were displayed with orange quadrates. Residues participate in PHR2 dimer interface were shown in cyan quadrates. Species are: *Oryza sativa* (Os), *Arabidopsis thaliana* (At), *Zea mays* (Zm), *Panicum hallii* (Ph), *Brachypodium distachyon* (Bd), *Ananas comosus* (Ac), *Glycine max* (Gm), *Sorghum bicolor* (Sb), *Prosopis alba* (Pa), *Vigna unguiculate* (Vu).

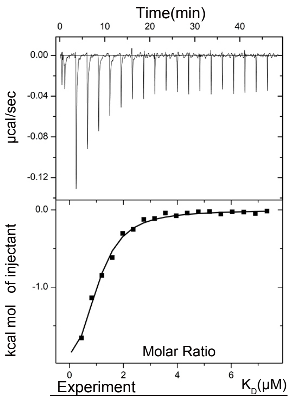

Experiment	K_D(μM)
$InsP_6$ VS $SPX1^{K147A}$	5.69±1.46

Supplementary Fig. 7 $SPX1^{K147A}$ has little effect on $InsP_6$ binding.

Binding affinity of $InsP_6$ with $SPX1^{1\text{-}259\ K147A}$ was measured by ITC.

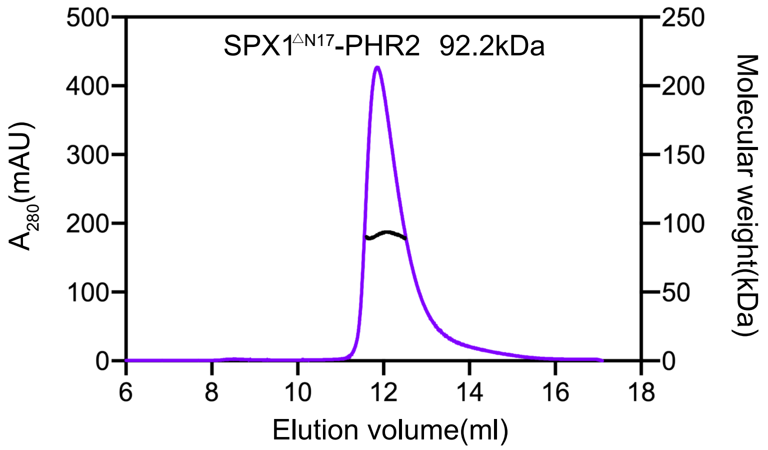

Supplementary Fig. 8 SPX1$^{\Delta17}$ fails to separate PHR2 dimer in the presence of InsP$_6$.

The molecular weight of SPX1$^{\Delta17}$-PHR2 complex in presence of InsP$_6$ measured by SEC-MALS assay is 92.2kDa, matching well with the theoretical molecular weight of two copies of SPX1$^{\Delta17}$ and two copies of PHR2 (96.8kDa).

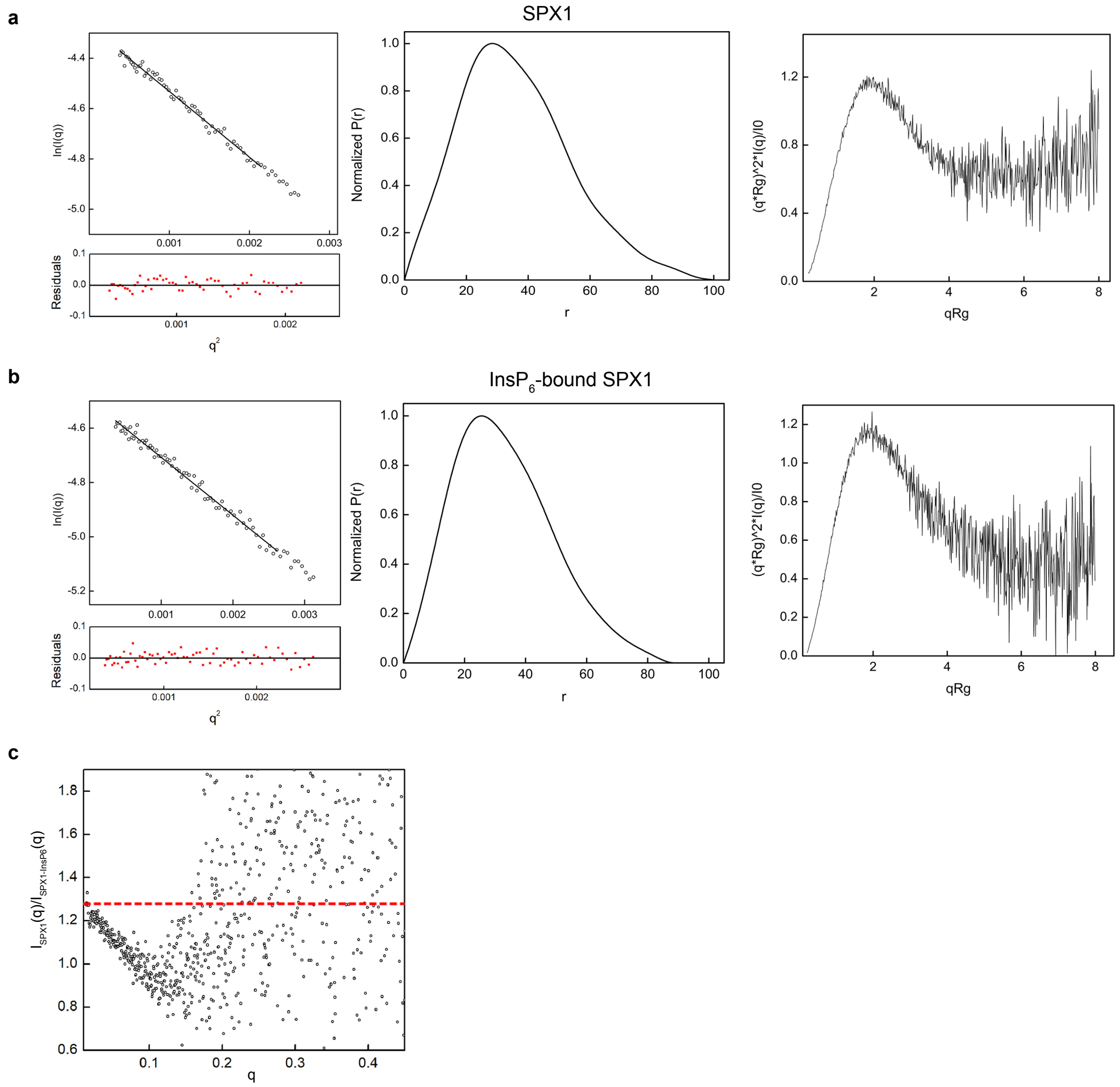

Supplementary Fig. 9 SPX1 and InsP$_6$-bound SPX1 appears to be different via SAXS.

a, b SAXS curves of SPX1 and InsP$_6$-bound SPX1. Guinier plot (left), Normalized P(r) analysis (middle) and dimensionless Kratky plot (right). The Guinier radius of gyration (Rg) and D_{max} for SPX1 is ~28.03 Å and 100 Å, larger than the values of InsP$_6$-bound SPX1 (25.33 Å and 89 Å respectively), calculated from their SAXS profiles. This result suggests that SPX1 is less compact than InsP$_6$-bound SPX1. Dimensionless Kratky plot shows a typical peak in both of SPX1 and InsP$_6$-bound SPX1 indicate that the samples were of folded conformations. **c** Scattering curve comparison between SPX1 and InsP$_6$-bound SPX1.

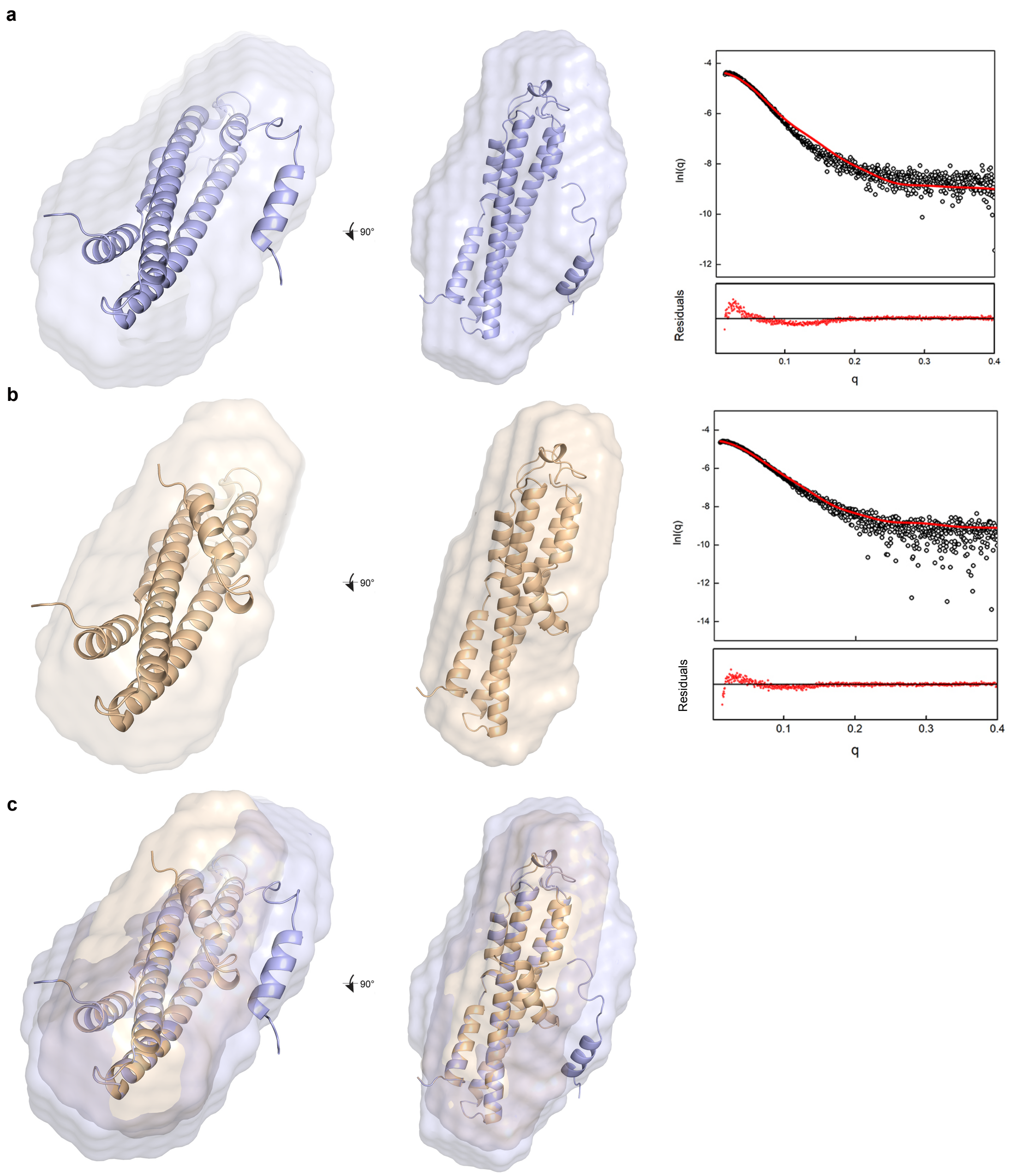

Supplementary Fig. 10 SAXS data for SPX1 and $InsP_6$-SPX1.

a Ab initio models of SPX1 built by DAMMIF and the fit of the SAXS envelope to the atomic structure model of SPX1 reconstituted by docking the helix α1 and the remaining part of $InsP_6$-SPX1 crystal structure into our SAXS bead model separately using SASREF (left). SPX1 atomic structure theoretical solution scattering curve (red) was compared to the experimental scattering curves (black) by FoXS (right) with a $\chi2$ of 5.2. **b** Ab initio molecular envelope of $InsP_6$-SPX1 showing the fitting of the $InsP_6$-SPX1 crystal structure (left). Scattering profile $InsP_6$-SPX1 atomic model was aligned to the experimental data and evaluated using FoXS with a $\chi2$ of 2.5 (right). **c** Superimposition of atomic structure models in **a** and **b,** and the respective molecular envelopes.

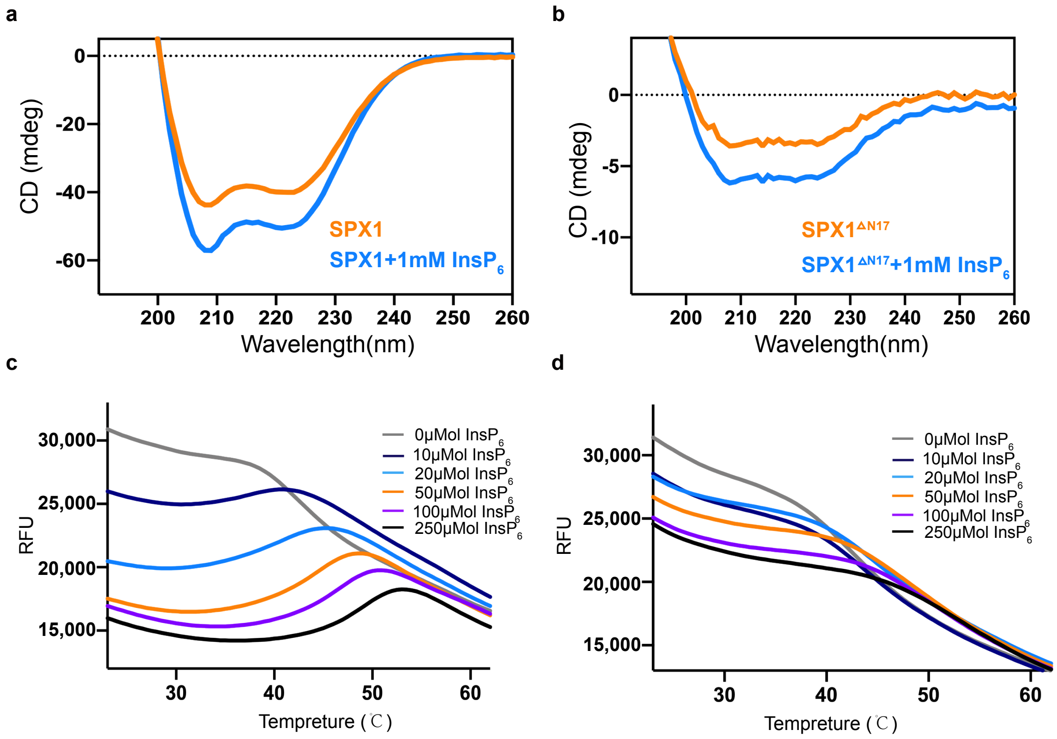

Supplementary Fig. 11 InsP$_6$ stabilizes SPX1 suggested by Circular Dichroism assay and thermal shift assay. **a, b** CD spectra of SPX1 and SPX1$^{\Delta N17}$ in the absence (orange) or presence (blue) of InsP$_6$ measured in the far-UV region (260-195 nm). The intensity of the two peaks at 208 nm and 220 nm increased in the presence of InsP$_6$, indicating the increased α-helical content in SPX1 significantly (a). InsP$_6$ didn't change the α-helical content of SPX1$^{\Delta N17}$ prominently (b). **c, d** Thermal melting profiles of SPX1 and SPX1$^{\Delta N17}$ at a series of concentration of InsP$_6$, (10 µM, dark blue; 20 µM, blue; 50 µM orange; 100 µM purple; 250 µM InsP$_6$ dark, (n= 3 technical replicates). The denaturation profile of SPX1 and SPX1$^{\Delta N17}$ (grey line) indicated that SPX1 has exposed hydrophobic regions in the native state. With increase of InsP$_6$, Tm value of SPX1 showed a significant change (c). InsP$_6$ only slightly affected Tm value of SPX1$^{\Delta N17}$ (d).

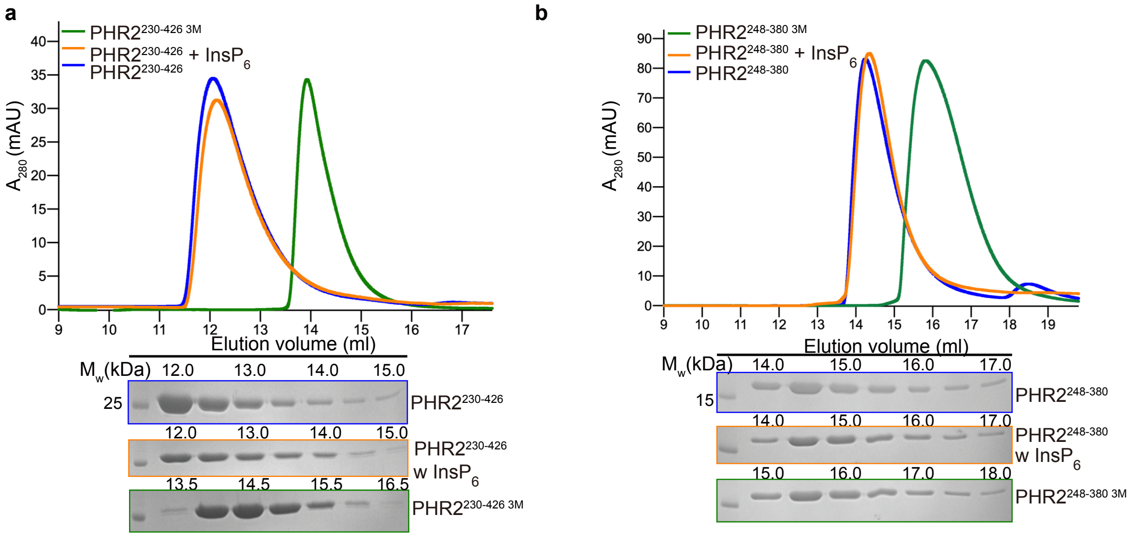

Supplementary Fig. 12 $InsP_6$ itself shows no effect on the dissociation of PHR2 dimer. a-b Elution volumes of PHR2$^{230\text{-}426}$ and PHR2$^{248\text{-}380}$ did not change in presence (purple) and absence (blue) of $InsP_6$. $InsP_6$ cannot dissociate the PHR2 dimer which eluted much earlier than the elution volume of monomeric PHR2 (PHR2^{3M}, orange). (Upper) Gel filtration profiles were color coded. (Lower) Coomassie-blue stained SDS-PAGE gels of peak fractions. The SEC assays were performed with Superdex 200 Increase 10/300 GL column. Experiments were independently repeated three times with similar results. Uncropped gel images are available as source data.

Supplementary Table 1 - Crystallography data collection and refinement statistics

	$InsP_6$-SPX1- Se-PHR2-derivative
**Data collection**	
Space group	$P2_12_12_1$
Cell dimensions	
a, *b*, *c* (Å)	65.24, 107.49,174.52
α, β, γ (°)	90, 90, 90
	Peak
Wavelength	0.97918
Resolution (Å)	50-2.6 (2.75-2.60)
R_{sym} or R_{merge}	0.108 (0.706)
I / σI	17.8 (3.4)
Completeness (%)	99.88 (99.92)
Redundancy	7.0 (6.8)
**Refinement**	
Resolution (Å)	2.60
No. reflections	38544
R_{work} / R_{free}	0.227/0.276
No. atoms	
Protein	7193
Ligand/ion	108/0
Water	17
B-factors	67.68
Protein	66.34
Ligand/ion	158.67/0
Water	57.17
R.m.s deviations	
Bond lengths (Å)	0.013
Bond angles (°)	1.78
Ramachandran statistics	
Favored	96.9%
Allowed	2.87%
Outliers	0.23%

This table describes the data collection, phasing and refinement statistics. Values in parentheses are for highest-resolution shell.

Supplemental Table 2. SAXS Data-collection and scattering-derived parameters

Data-collection parameters		
Instrument	BL19U2 beamline, equipped with a Pilatus 2M Detector, SSRF	
Beam geometry	Scatterless Slits	
Wavelength (Å)	1.033	
q range ($Å^{-1}$)	0.01-0.35	
Exposure time (sec)	1.0 per frame	
Concentration range (mg ml^{-1})	1-2	
Temperature (K)	296	
**Guinier analysis**	SPX1	$InsP_6$-bound SPX1
R_g (Å)	28.03± 0.57	25.33±0.23
I(0) (cm^{-1})	0.014 ± 7.4e-05	0.011±5.2e-05
**GNOM**		
R_g (Å)	28.46	26.05
I(0) (cm^{-1})	0.01	0.01
D_{max} (Å)	100	89
Software employed		
Primary data reduction		BioXTAS RAW
Ab initio analysis		DAMMIF
Rigid-body modelling		SASREF
Computation of model intensities		FoXS
Three-dimensional graphics representations		PyMOL

Supplementary Table 3. Oligonucleotide primers used in this research.

Primer name	Sequence (5'-3')
SPX1-F	AAAACCTCTACTTCCAATCGATGAAGTTTGGGAAGAGCCTG
SPX1-259-R	CCACACTCATCCTCCGGTCATCCGCTTCGGATCTCCTT
SPX1-198-R	CCACACTCATCCTCCGGTCATTCGTTTGATGGTAGAAG
SPX1-198-T4L-F	CTTCTACCATCAAACGAAATTTTTGAAATGCTG
SPX1-198-T4L-R	CAGCATTTCAAAAATTTCGTTTGATGGTAGAAG
T4L-R	CCACACTCATCCTCCGGTCAATATGCATCCCAGGT
SPX-△N17-F	AAAACCTCTACTTCCAATCGTGGCGGGACAAGTTCTTGTCG
SPX1-Y25F-K29A-F	AAGTTCTTGTCGTTCAAGGATCTCGCAAAGCGGCTCAAG
SPX1-Y25F-K29A-R	CTTGAGCCGCTTTGCGAGATCCTTGAACGACAAGAACTT
SPX1-K151A-F	TTAGTTAAGATTCTCAAGGCATATGACAAGAGGACT
SPX1-K151A-R	AGTCCTCTTGTCATATGCCTTGAGAATCTTAACTAA
SPX1-K147A-F	ACCGGATTAGTTGCAATTCTCAAGAAGTATGACAAG
SPX1-K147A-R	CTTGTCATACTTCTTGAGAATTGCAACTAATCCGGT
SPX-TRX-F	CCATCAAACGAACTGTCTGTATCGAGTATGAGCGATAAAATTATTCACCTGACT
SPX-TRX-R	AGTCAGGTGAATAATTTTATCGCTCATACTCGATACAGACAGTTCGTTTGATGG
TRX-R	CCACACTCATCCTCCGGTTAGGCCAGGTTAGCGTCGAGGAA
PSAT-6-SPX1-F	CGAGCTCAAGCTTCGAATTCATGAAGTTTGGTAAGAGTCTC
PSAT-6-SPX1-R	CGGGCCCGCGGTACCGTTTGGCTTCTTGCTCCAACAA
SPX1-q-F	TATATGAAGGGCACGGTCGC
SPX1-q-R	GCCCTGGAGAGGTGGTAATG
IPS1-q-F	GGGCACACTCCACATTATCCA
IPS1-q-R	CACCATTAGCCCACGCTCTA
PT2-q-F	CGCCCAAGCGTGATGTCATA
PT2-q-R	ACCCCACAAATCCACAACTG
Actin-q-F	CAACACCCCTGCTATGTACG
Actin-q-R	CATCACCAGAGTCCAACACAA
SPX1-pro-F	ACGACGGCCAGTGCCAAGCTTGTTCTTGATATATGCG
SPX1-pro-R	GAGCTCGGTACCCGGGGATCCCCTCAACAACAAGAGA
SPX1-cds-F	CTCTTGTTGTTGAGGGGATCCATGAAGTTTGGGAAGA
SPX1-cds-R	GGGAAATTCGAGCTCGGTACCTCATTTGGCGGCCTGC
SPX1-E89A-F	AAGTTCAACTCCTTCTTCGTCGCAAAGGAGGAGGAATACATCATC
SPX1-E89A-R	GATGATGTATTCCTCCTCCTTTGCGACGAAGAAGGAGTTGAACTT
SPX1-E92A-F	TCCTTCTTCGTCGAGAAGGAGGCAGAATACATCATCCGCCAGAAG
SPX1-E92A-R	CTTCTGGCGGATGATGTATTCTGCCTCCTTCTCGACGAAGAAGGA
SPX1-I96A-F	GAGGAGGAATACATCGCACGCCAGAAGGAGCTG
SPX1-I96A-R	CAGCTCCTTCTGGCGTGCGATGTATTCCTCCTC
SPX1-K99A-F	GAGGAATACATCATCCGCCAGGCAGAGCTGCAGGACAGGGTGGCG
SPX1-K99A-R	CGCCACCCTGTCCTGCAGCTCTGCCTGGCGGATGATGTATTCCTC
SPX1-E100A-F	GAATACATCATCCGCCAGAAGGCACTGCAGGACAGGGTGGCGAGG
SPX1-E100A-R	CCTCGCCACCCTGTCCTGCAGTGCCTTCTGGCGGATGATGTATTC

SPX1-V125A-F	GTGCGCAAGGAGATCGCAGACTTCCATGGCGAG
SPX1-V125A-R	CTCGCCATGGAAGTCTGCGATCTCCTTGCGCAC
SPX1-V132A-F	TTCCATGGCGAGATGGCACTGCTCGAGAACTAC
SPX1-V132A-R	GTAGTTCTCGAGCAGTGCCATCTCGCCATGGAA
SPX1-E135A-F	CATGGCGAGATGGTGCTGCTCGCAAACTACAGCGCCCTCAACTAC
SPX1-E135A-R	GTAGTTGAGGGCGCTGTAGTTTGCGAGCAGCACCATCTCGCCATG
SPX1-R161A-F	AAGAGGACTGGGGCTCTGATCGCACTGCCTTTCATCCAGAAAGTG
SPX1-R161A-R	CACTTTCTGGATGAAAGGCAGTGCGATCAGAGCCCCAGTCCTCTT
SPX1-Y180A-F	TTCTTCACCACTGACCTCCTGGCAAAGCTTGTGAAACAGTGTGAG
SPX1-Y180A-R	CTCACACTGTTTCACAAGCTTTGCCAGGAGGTCAGTGGTGAAGAA
PHR2-230-F	AAAACCTCTACTTCCAATCGGCAGTTGCTATACCCTCGCCC
PHR2-426-R	CCACACTCATCCTCCGGTTATCTGTCACCTGATTCTGT
PHR2-L348A-F	GAACTCCAAAAGAGGGCACATGAACAGCTTGAG
PHR2-L348A-R	CTCAAGCTGTTCATGTGCCCTCTTTTGGAGTTC
PHR2-L358A-F	GAGATCCAAAGAAGTGCACAGCTGAGAATTGAG
PHR2-L358A-R	CTCAATTCTCAGCTGTGCACTTCTTTGGATCTC
PHR2-I362A-F	AGTTTGCAGCTGAGAGCAGAGGAGCAAGGGAAG
PHR2-I362A-R	CTTCCCTTGCTCCTCTGCTCTCAGCTGCAAACT
PHR2-L348A/L358A/I362A-F	CATGAACAGCTTGAGATCCAAAGAAGTGCACAGCTGAGAGCAGAGGAGCAAGGGAAG
PHR2-L348A/L358A/I362A-R	TGCTCTCAGCTGTGCACTTCTTTGGATCTCAAGCTGTTCATGTGCCCTCTTTTGGAG
PHR2-L344R/Q351R/Q355R-F	AGACAAAAGAGGCTTCATGAAAGACTTGAGATCAGAAGAAGTTTGCAGCTG
PHR2-L344R/Q351R/Q355R-R	TCTGATCTCAAGTCTTTCATGAAGCCTCTTTTGTCTTTCTAACTGGAGACG
PHR2-248-F	AAAACCTCTACTTCCAATCGCGAATGAGATGGACTCCTGAA
PHR2-380-R	CCACACTCATCCTCCGGTTACCCAGGTATGCACTGCTG
PHR2-V263M-F	CGAATGAGATGGACTCCTGAACTTCATGAGCGCTTTGTAGATGCTATGAATCTACTTGGTGGCAGT
PHR2-L278M-F	GAAAAAGCTACTCCCAAGGGTGTGATGAAGCTAATGAAGGCAGAC
PHR2-L278M-R	GTCTGCCTTCATTAGCTTCATCACACCCTTGGGAGTAGCTTTTTC
PHR2-L295M-F	TATCATGTTAAAAGTCACATGCAGAAATACAGAACAGCT
PHR2-L295M-R	AGCTGTTCTGTATTTCTGCATGTGACTTTTAACATGATA
PHR2-L340M-F	CTCACTGAGGCATTGCGTATGCAGTTAGAACTCCAAAAG
PHR2-L340M-R	CTTTTGGAGTTCTAACTGCATACGCAATGCCTCAGTGAG
PHR2-R248A-F	AAAACCTCTACTTCCAATCGGCAATGAGATGGACTCCTGAA
PHR2-E257A-F	AAAACCTCTACTTCCAATCGCGAATGAGATGGACTCCTGAACTTCATGCACGCTTTGTA
PHR2-H294A-F	ACCATTTATCATGTTAAAAGTGCACTTCAGAAATACAGAACAGCT
PHR2-H294A-R	AGCTGTTCTGTATTTCTGAAGTGCACTTTTAACATGATAAATGGT
PHR2-K297A-F	CATGTTAAAAGTCACCTTCAGGCATACAGAACAGCTCGATACAGA
PHR2-K297A-R	TCTGTATCGAGCTGTTCTGTATGCCTGAAGGTGACTTTTAACATG
PHR2-R302A-F	CTTCAGAAATACAGAACAGCTGCATACAGACCAGAATTGTCTGAA
PHR2-R302A-R	TTCAGACAATTCTGGTCTGTATGCAGCTGTTCTGTATTTCTGAAG
PHR2-L342A-F	GCATTGCGTCTCCAGGCAGAACTCCAAAAGAGG
PHR2-L342A-R	CCTCTTTTGGAGTTCTGCCTGGAGACGCAATGC
PHR2-H349A-F	TTAGAACTCCAAAAGAGGCTTGCAGAACAGCTTGAGATCCAAAGA
PHR2-H349A-R	TCTTTGGATCTCAAGCTGTTCTGCAAGCCTCTTTTGGAGTTCTAA

PHR2-L352A-F	AGGCTTCATGAACAGGCAGAGATCCAAAGAAGT
PHR2-L352A-R	ACTTCTTTGGATCTCTGCCTGTTCATGAAGCCT
PHR2-E353A-F	AAGAGGCTTCATGAACAGCTTGCAATCCAAAGAAGTTTGCAGCTG
PHR2-E353A-F	CAGCTGCAAACTTCTTTGGATTGCAAGCTGTTCATGAAGCCTCTT
PHR2-R356A-F	CATGAACAGCTTGAGATCCAAGCAAGTTTGCAGCTGAGAATTGAG
PHR2-R356A-R	CTCAATTCTCAGCTGCAAACTTGCTTGGATCTCAAGCTGTTCATG
PHR2-L360A-F	CAAAGAAGTTTGCAGGCAAGAATTGAGGAGCAA
PHR2-L360A-R	TTGCTCCTCAATTCTTGCCTGCAAACTTCTTTG
PHR2-E363A-F	CAGCTGAGAATTGCAGAGCAAGGGAAGTGCCTTCAGATGATGCTCGAGCAGCAGTGCATACCTGGG
PHR2-E363A-R	CCCAGGTATGCACTGCTGCTCGAGCATCATCTGAAGGCACTTCCCTTGCTCTGCAATTCTCAGCTG
PSAT-6-PHR2-F	CGAGCTCAAGCTTCGAATTCATGGAGAGAATAAGCACCAAT
PSAT-6-PHR2-R	CGGGCCCGCGGTACCGTCTGTCACCTGATTCTGTTTG
PHR1-227-F	AAAACCTCTACTTCCAATCGCGAATGCGTTGGACGCCAGAG
PHR1-358-R	CCACACTCATCCTCCGGTCAACCAGAGTTTTGCTTCTC
