# Supplementary material for: Mechanism of phosphate sensing and signaling revealed by rice SPX1-PHR2 complex structure
Source: Nat Commun. 2021 Dec 2;12:7040. doi: 10.1038/s41467-021-27391-5 (PMC8639918; doi:10.1038/s41467-021-27391-5)
Supplement: Supplementary file 3 — Reporting Summary [file 41467_2021_27391_MOESM3_ESM.pdf]

nature portfolio

Corresponding author(s): Weiman Xing

Last updated by author(s): Oct 30, 2021

Reporting Summary

Nature Portfolio wishes to improve the reproducibility of the work that we publish. This form provides structure for consistency and transparency in reporting. For further information on Nature Portfolio policies, see our Editorial Policies and the Editorial Policy Checklist.

Statistics

For all statistical analyses, confirm that the following items are present in the figure legend, table legend, main text, or Methods section.

n/a	Confirmed	
☐	☒	The exact sample size (*n*) for each experimental group/condition, given as a discrete number and unit of measurement
☐	☒	A statement on whether measurements were taken from distinct samples or whether the same sample was measured repeatedly
☐	☒	The statistical test(s) used AND whether they are one- or two-sided<br>*Only common tests should be described solely by name; describe more complex techniques in the Methods section.*
☒	☐	A description of all covariates tested
☒	☐	A description of any assumptions or corrections, such as tests of normality and adjustment for multiple comparisons
☐	☒	A full description of the statistical parameters including central tendency (e.g. means) or other basic estimates (e.g. regression coefficient) AND variation (e.g. standard deviation) or associated estimates of uncertainty (e.g. confidence intervals)
☐	☒	For null hypothesis testing, the test statistic (e.g. *F*, *t*, *r*) with confidence intervals, effect sizes, degrees of freedom and *P* value noted<br>*Give P values as exact values whenever suitable.*
☒	☐	For Bayesian analysis, information on the choice of priors and Markov chain Monte Carlo settings
☒	☐	For hierarchical and complex designs, identification of the appropriate level for tests and full reporting of outcomes
☒	☐	Estimates of effect sizes (e.g. Cohen's *d*, Pearson's *r*), indicating how they were calculated

Our web collection on statistics for biologists contains articles on many of the points above.

Software and code

Policy information about availability of computer code

Data collection	The crystal data sets were collected at beamlines BL17U1 and BL19U1 at the Shanghai Synchrotron Radiation Facility and processed with the software XDS.<br>The small angle X-ray scattering data were collected at the BL19U2 beamline at Shanghai Synchrotron Radiation Facility.
Data analysis	Structure determination was carried out using Phenix 1.18_3816. The model building was done using Coot-0.8.9.<br>ITC data was collected and processed by Microcal PEAQ-ITC analysis software and Origin 7.0.<br>The multiple angle light scattering analysis was performed on an AKTA Pure system (GE Healthcare) coupled with a static light scattering detector (miniDawn, Wyatt) and a differential refractive index detector (Optilab, Wyatt).<br>Real-time qPCR was performed by CFX96 real-time PCR detection system (Bio-Rad).<br>LUC luminescence was measured with a plate reader (Perkin Elmer, USA).<br>CD data was collected and processed by Chirascan™-plus CD.<br>Thermal shift assays were conducted by CFX Connect Real-Time PCR detection system (Bio-Rad).<br>SAXS data was analysis by BioXTAS RAW, DAMMIF, FoXS and SASREF.

For manuscripts utilizing custom algorithms or software that are central to the research but not yet described in published literature, software must be made available to editors and reviewers. We strongly encourage code deposition in a community repository (e.g. GitHub). See the Nature Portfolio guidelines for submitting code & software for further information.

Data

Policy information about availability of data

All manuscripts must include a data availability statement. This statement should provide the following information, where applicable:

- Accession codes, unique identifiers, or web links for publicly available datasets
- A description of any restrictions on data availability
- For clinical datasets or third party data, please ensure that the statement adheres to our policy

Structural coordinates and structural factors have been deposited in the Protein Data Bank under accession number 7E40 [https://www.rcsb.org/structure/unreleased/7E40]. Source data are provided with this paper.

Field-specific reporting

Please select the one below that is the best fit for your research. If you are not sure, read the appropriate sections before making your selection.

☒ Life sciences ☐ Behavioural & social sciences ☐ Ecological, evolutionary & environmental sciences

For a reference copy of the document with all sections, see nature.com/documents/nr-reporting-summary-flat.pdf

Life sciences study design

All studies must disclose on these points even when the disclosure is negative.

Sample size	Sample sizes were chosen based on the typical number of replicates reported for similar studies.
Data exclusions	No data were excluded from the analysis.
Replication	Indicated experiments were repeated at least 3 times.
Randomization	Samples were selected randomly.
Blinding	The growth experiments of rice wt and mutants were done blindly.

Reporting for specific materials, systems and methods

We require information from authors about some types of materials, experimental systems and methods used in many studies. Here, indicate whether each material, system or method listed is relevant to your study. If you are not sure if a list item applies to your research, read the appropriate section before selecting a response.

Materials & experimental systems

n/a	Involved in the study	
☒	☐	Antibodies
☒	☐	Eukaryotic cell lines
☒	☐	Palaeontology and archaeology
☒	☐	Animals and other organisms
☒	☐	Human research participants
☒	☐	Clinical data
☒	☐	Dual use research of concern

Methods

n/a	Involved in the study	
☒	☐	ChIP-seq
☒	☐	Flow cytometry
☒	☐	MRI-based neuroimaging
